# Health-care seeking behaviour and the use of traditional medicine among persons with type 2 diabetes in south-western Uganda: a study of focus group interviews

**DOI:** 10.11604/pamj.2015.20.76.5497

**Published:** 2015-01-29

**Authors:** Fortunate Atwine, Sally Hultsjö, Björn Albin, Katarina Hjelm

**Affiliations:** 1 School of Health and Caring Sciences, Linnaeus University, Växjö, Sweden; 2 Department of Nursing, Mbarara University of Science and Technology (MUST), Mbarara, Uganda; 3 Department of Social and Welfare Studies, University of Linköping, Campus Norrköping, Sweden

**Keywords:** Complementary alternative medicine, diabetes mellitus, health-care seeking behaviour, traditional healers, nursing, Uganda

## Abstract

**Introduction:**

Health-care seeking behaviour is important as it determines acceptance of health care and outcomes of chronic conditions but it has been investigated to a limited extent among persons with diabetes in developing countries. The aim of the study was to explore health-care seeking behaviour among persons with type 2 diabetes to understand reasons for using therapies offered by traditional healers.

**Methods:**

Descriptive study using focus-group interviews. Three purposive focus-groups were conducted in 2011 of 10 women and 7 men aged 39–72 years in Uganda. Data were collected through semi-structured interviews and qualitatively analysed according to a method described for focus-groups.

**Results:**

Reasons for seeking help from traditional healers were symptoms related to diabetes such as polydipsia, fatigue and decreased sensitivity in lower limbs. Failure of effect from western medicine was also reported. Treatment was described to be unknown extracts, of locally made products taken as herbs or food, and participants had sought help from different health facilities with the help of relatives and friends.

**Conclusion:**

The pattern of seeking care was inconsistent, with a switch between different health care providers under the influence of the popular and folk sectors. Despite beliefs in using different healthcare providers seeking complementary and alternative medicine, participants still experienced many physical health problems related to diabetes complications. Health professionals need to be aware of the risk of switches between different health care providers, and develop strategies to initiate health promotion interventions to include in the care actors of significance to the patient from the popular, folk and professional sectors, to maintain continuity of effective diabetes care.

## Introduction

Health-care seeking behaviour is important because it is one of the factors determining the acceptance of health care and outcomes, especially in chronic conditions [1], but it has only been investigated to a limited extent among persons with diabetes in developing countries. People decide whom to consult and when, whether to comply with treatment or change health care service providers using the existing local health care systems. Health care can be sought in different sectors in society [2], among family and friends in the popular sector, from traditional healers (sacred or secular) in the folk sector, and/or from health care staff in the professional sector of different health care institutions. A common behaviour is to start with self-care, and if the health problem persists, recourse is made to the popular sector, such as family, friends or neighbours before help from medical care in the professional health sector or the folk sector [2]. Where to turn is related to the health care system in the society and other socioeconomic factors: costs for transport, consultations and treatment. An exception is a study in Uganda which revealed that health care was mainly sought from doctors and nurses in the professional health sector and that perceived failure of health care to manage the disease or diabetes-related complications led many to seek traditional healers in the folk sector [3]. Despite the broad use of traditional medicine in Uganda, there are few data on care seeking behaviour and diabetes management using traditional healers.

Diabetes mellitus (DM) is a chronic, progressive disease with micro- and macrovascular complications likely to develop over time due to poor glycaemic control [4], which requires adaptation to new ways of living to control the disease through self-care and medical treatment including regular follow-up [5]. However, switches between different health care sectors may interrupt glycaemic control and negatively affect health [6]. DM has become a global concern, due to the increasing incidence of type 2 diabetes mostly affecting people in Africa and Asia [4]. It is known that traditional healers have for many years served 80–90% of the African population in primary health care [7], including those with diabetes [8]. Reasons for using traditional healers to manage diabetes were reported in a study from eastern Uganda: limited resources, poor availability of health facilities, and affordability constraints to diabetes drugs in the professional health care sector [9]. On the other hand, delays in diagnosis due to health care seeking in Tanzania were attributed to patients’ poor knowledge about diabetes and associated misconceptions of the presenting signs [1]. Underlying living conditions such as the affordability of drugs, food, equipment for self-monitoring of blood glucose and different gender roles also determine beliefs about health and illness and affect health-related practices including health-care seeking behaviour [10].

While in developed countries diabetes care is largely sought in medical centres in primary health care it is different in Africa, where care is sought from public hospitals, private and traditional healers in the folk sector [8]. Diabetes care in Uganda is run within government referral hospitals, private not-for-profit (faith-based) health facilities and private medical care on recommendations by physicians. Despite the development of outpatient diabetes clinics in government referral hospitals, there is a mismatch between a high number of patients and the availability of resources to satisfy their needs [11]. The situation is very challenging, so that patients are forced to take their own decisions in searching for convenient health care [12]. Besides this formal organization there are also traditional and complementary practices such as herbalists, traditional birth attendants, bone setters and spiritual healers [13]. Problems of diabetes management in Sub-Saharan Africa include late and poor clinic attendance, delayed diagnosis and poor quality of services [3]. Inadequate health-care seeking behaviour is one of the determinants of poor health in persons with diabetes [14].

Diabetes mellitus requires adaptation to a newly learned lifestyle to control the disease [3]. One of the best strategies is to provide individualized health care through a supportive-educative nursing system [15]. This helps a patient master her/his condition, adhering to the adjusted diet and lifestyle, checking blood sugar and drug administration if necessary for sustainable self-care maintenance [16]. However, the complexity of diabetes may reverse the process and cause a switch from formal practices to the use of healers in the folk sector, since this is deeply rooted in culture and more congruent personal values, beliefs and philosophy concerning health and life [17]. Understanding human behaviour in health-care seeking among persons with diabetes gives information needed by health professionals to provide effective treatment that will have positive outcomes. There is a knowledge gap about how persons with diabetes treat their conditions using local available health care systems in Uganda. The aim of the study therefore, sought to explore health-care seeking behaviour among persons with type 2 diabetes, to understand reasons for using therapies offered by traditional healers.

## Methods

### Design

A qualitative descriptive study design, with data collected by focus-group interviews, was used. Focus-group interviews were preferred in order to generate rich data and capture people's experiences as the field had not been explored, and as this method stimulates interaction between participants that might reveal both conscious and unconscious beliefs and experiences [18]. Group interaction is supposed to support the participants in remembering events, beyond the answers of a single interviewee.

### Participants

Participants were recruited through purposive sampling of information-rich individuals [19] from three traditional healers’ facilities in south-western Uganda. The managers of the health facilities invited clients visiting the clinic to participate. People were eligible to participate if they had been diagnosed with type 2 diabetes mellitus, were >18 years old, were able to talk for themselves, had no known psychiatric disorder and freely consented to participate. Ten females and seven males, aged 39–72 years (Md 59), were recruited (Table 1).

**Table 1:** Characteristics of the studied population

Variables	N = 17
**Gender (n)**	
Women	10
Men	7
**Age (year)**^1^	59 (39–72)
Duration of DM (years) 1	3 (0.6–14)
**Treatment (n)**	
Diet	0
Oral agents	10
Insulin	4
Alternative medicine	3
**Marital status (n)**	
Married/cohabitant	14
Widow/er	3
**Educational level (n)**	
Illiterate	4
Primary school	12
Secondary school	1
**Socio-economic status (n)**	
Low	17
Middle	0
High	0
**Self-reported complications related to diabetes (n)**	
Eye	5
Heart	6
Feet/lower extremity	9

1 Values are median (range)

### Data collection

**Figure 1:**
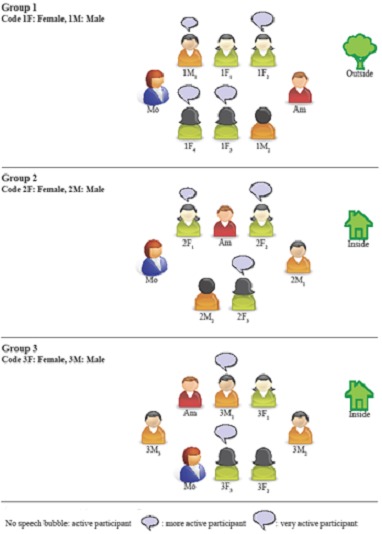
The interaction in the groups was lively.

Data were collected between February and April 2011 in focus-group interviews. Three focus-groups with five to six participants in each were held, giving a total number of 17 participants. The interviews were led by a bilingual moderator, a nurse (first author), and an assistant moderator tape-recorded the sessions and took field notes. The group interviews were held in natural and quiet places to enhance good interaction. Sitting arrangements and group interactions (which were lively) are described in Figure 1.

The interview guide provided successive interviews and was built on information obtained from a previous study [6]; it was peer-reviewed by nurses and general practitioners working in diabetes care. A pilot test was done (not included in the study) on two women and led to minor corrections in the interview guide. Questions were logically posed following the semi-structured interview guide from demographic and medical data to specific questions in relation to the purpose of the study, allowing flexibility and probing for clarity [18]. The main questions were: What problems have you sought help for? What have they treated with according to the above problems? What effects does the treatment have? Have you sought for healthcare elsewhere? If yes, where? How do you get advice for seeking help?

The interactive nature of the interviews allowed the moderator to ask questions to ensure that participants were clearly understood. A disadvantage of focus groups is that the informants may influence each other or try to impress each other [18]. To avoid this, the moderator noted and reflected on the group dynamics, and actively listened to ensure the responses answered the questions. All interviews lasted 90–120 minutes in free and flowing discussions using the local Runyankore dialect, were audiotaped and transcribed verbatim. The interviews were then translated into English, and then back-translated by the bilingual moderator (first author). Runyankore is one of about 40 different indigenous languages in Uganda [20] belonging to the Bantu languages, which are spoken in most of the southern half of the country. The Bantu languages are mutually comprehensible.

### Ethical considerations

The study was approved by Faculty Research Ethical Committee (FREC) of the University in the area and was carried out in accordance with the Helsinki Declaration [21]. Verbal informed consent was obtained from participants before proceeding with interviews.

### Data analysis

Data were analysed as described for focus groups, [18]. Collection of data and analysis proceeded simultaneously and saturation was judged to have occurred after the third group interview when there was no new information forthcoming. It was a transcript-based analysis. All transcripts were first read several times to obtain a sense of the whole. The analyses were based on openness to variation in the data and a search for patterns, regularities and contradictions by constantly comparing different statements by participants. By reviewing each line of the text, meaning units were identified and coded. Phrases with similar meaning were condensed into common sub-categories. Categories were merged that linked the text to answer research questions. The model for health-care seeking behaviour [2] was introduced to give the main analytical framework in the development of categories, as previously described [6, 10], that health care can be sought from three sectors: (1) popular among family/friends, (2) folk from traditional healers, (3) professionals in the formal health care system. The names of categories were as close to the terms in the original text as possible. Then descriptive summaries were made from each category. For an example of data analysis see Table 2.

**Table 2:** An example of data analysis concerning reasons for seeking help

Illuminations	Code	Subcategory No of statements	Category No of statements
…experiencing much heat, and bodily pain especially the feet	Heat/pain in body and feet	Painful Limbs (6)	Painful Limbs (6)
…and a sensation of pins and needles in my feet	Pins and needles sensation	Decreased sensitivity (3)	
…because of excessive thirst and passing urine many times especially at night	Excessive thirst, passing urine many times at night	Nykturia/polydipsia (3)	Symptoms related to poor glycaemic control (13)
…if I do the tongue gets dry… then I get excessive thirst. The throat becomes very dry and I feel it almost giving away tearing	Excessive thirst feeling dry throat	Polydipsia (3)	
…and passing urine many times	Passing urine many times	Polyuria (5)	
…at times I don't see well I feel dizzy	Do not see well, feel dizzy	Drowsy (2)	
…I was…on a motor-bike, something unclear attacked me and I fell down	Something unclear attacked and I fell down	Fainting (1)	
…the noise is like SSSeee andthereafter, I feel pounding headache	Pounding headache	Headache (6)	
…sweet taste I get abdominal pain	Get abdominal pain	Abdominal pain (4)	Different body pains (11)
…I have pain in my back…	Pain in the back	Backache (1)	
…I have to take medicine for…high blood pressure	High blood pressure	Hypertension (6)	
…I have problems with my sight...first went to hospital seeking for help for my eyes for fear of loosing my sight	Problems with sight	Poor sight/impaired vision (3)	Other diseases (13)
…not generally feeling well, I have heart palpitations	Heart palpitation	Cardiac problem (2)	
I went to the hospital and they told me I had high blood pressure and diabetes I have poor health generally… the heart and I am worried of HIV	Worried of HIV	HIV (1)	
I have abdominal pain, high blood pressure and known gastric ulcers	Gastric ulcers	Gastric ulcers (1)	
…I had complications of any kind. I used to take western medicine but it was if the condition was not improving	Condition not improving on western medicine	Western medicine not effective (3)	Failing western medicine (3)
…my friends who suffered the same condition told me that you don't have to stop taking the given hospital medicine. If you take both you stand a better chance of getting better	Combination of western and traditional medicine	Combination of treatment (1)	Complementary medicine (1)

### Rigour

To increase the trustworthiness of findings, dependability was ensured through investigator triangulation [19]. The texts were analysed independently and compared by three more researchers experienced in diabetes care and nursing science, and high agreement was reached. Confirmability refers to the objectivity of the data [19]. This was achieved by accurately answering the research questions, probing for clarity where necessary and some of the illuminations of participants are manifested in the findings. As for transferability, this study was not intended to generalize the findings, but to give a description of persons with diabetes using traditional healing to better understand their situation. However, it is up to the reader to consider the rigorous methods and participant characteristics and use or transfer the findings to another similar environment [19]. Fairness was ensured as different views, even if they were in the minority, were all presented.

## Results

Ten females and seven males, aged 39–72 (Md 59) were recruited (Table 1). Most were treated with a combination of oral agents or insulin and complementary alternative medicine and had a short duration of DM. Most were married, low educated and had occupations classified as low socio-economic status.

### Reasons for seeking help from traditional healers

The main reasons for seeking help from traditional healers were symptoms related to diabetes, either poor glycaemic control or complications related to the disease and problems in the lower limbs such as pain or decreased sensitivity (see Table 2). In some cases the respondents stated failure of effect from western medicine or seeking alternative medicine:

…the legs get numb, feeling tight…as if blood is not flowing… the hands also in the middle parts get numb, losing the sense of feeling…and coldness in the feet proceeds…the tongue becomes very dry…feeling excessive thirst and the throat becomes dry feeling it almost giving way or tearing…I feel the signs of diabetes like headache, numbness in my limbs and I have taken a long time without checking for diabetes. I now have high blood pressure, but the main thing is taking anything with a sweet taste it gives me abdominal pain… I failed to take diabetes tablets from the main hospital. I used to vomit, lose appetite, get hungry and feel as if I was going to die; there was no effect at all…my brother-in-law used to bring medicine in a 5-litre container from a man in Kampala, Kayunga in Mukono district but now my brother in law lost contact with him. He used to meet him in Kampala so my friends have directed me here, those who have been here as well (1f3).

### Treatment by traditional healers

The treatments given by the traditional healers were mainly described as unknown extracted medicine or in some cases as home-made products taken as medicine or food such as local herbs or local food:

“…medicine is already extracted in bottles. We do not know it (3m3).

…we are taught some conditions that affect us and how you can prevent them. We are advised to take raw food to cleanse the blood or boost the appetite. For example extracts from avocado leaves improve appetite and eucalyptus leaves reduce blood sugar (3f2).

The perceived effect of treatment by traditional healers was mainly symptom relief; a few talked about perceived reduction of blood sugar or restoration of body function. Some said the treatment had no or unknown effect and hoped it would work while others discussed worsened well-being on complementary medicine: …for me blood sugar came down, but sometimes taking herbal medicine you don't continue with it after feeling better. You feel the signs of raised blood sugar going down. For us who have lived with diabetes for long you can feel whether blood sugar is high or low, for example when blood sugar is high you feel sleepy, weak, no hunger and you start passing urine more often than usual and feeling excessive thirst. I tell you from this experience, in emergency taking guava leaves and chewing them two or three times a day if you feel that blood sugar is high and it will go down. (2f3)

…I have just started, I don't know the outcome yet, but I will keep coming. (2f1)

…when I take both medicines at the same time from the hospital and what I get here I get dizziness and general weakness. (3m1)

…at times the situation gets worse so you need to go to the hospital (3m3).

### Searching for help elsewhere

Most participants met at the traditional healers said they had sought help elsewhere, mainly from the professional health care sector in governmental hospitals, health centres, and private clinics. Some combined the professional health care sector with seeking help from governmental hospitals and the folk sector with traditional medicine, and in one case all health care sectors – professional, folk and popular – were used:

…I started with high blood pressure and got treatment from X hospital in Y district, Z health centre and at Dr. X‘s private clinic in …town. (1f4)

… I have high blood pressure and diabetes. If I am not here (traditional healer) I go to receive treatment from the main hospital of X. (2f1)

…I have lived with diabetes for 14 years now. I cannot tell it all, because I have visited many places, friends and other people have advised me to take herbs, fruits other food which I take to keep my blood sugar down. I go to the hospital for diabetic treatment. (2f2)

Most participants said they had received advice about where to search for help from people in the popular sector: relatives, friends or other senior patients. Some use mass media, especially listening to radio programmes. One person combined listening to the radio with experienced patients in the popular sector, and another combined friends, patients and health care staff in the popular and professional sectors.

…my daughter helps me. She brought me from X hospital in Y district. This is my second time coming here from Dr. Z's private clinic in town. (1f2)

…I got help from friends and other patients to get the same treatment that has worked for them.

(1f1) …from radio and other patients. (1m2)

…when you are searching for a treasure you look everywhere not leaving any stone unturned. I have visited many health facilities, using many medicinal and food plants with the help of friends, patients, nurses and doctors (2f2).

## Discussion

This study explored health-care seeking behaviour among persons with diabetes and their experiences in using traditional healers in the folk sector. It offered a unique opportunity to describe people's experiences of self-care initiatives undertaken to cope with challenges associated with diabetes in a developing country.

Specific reasons for seeking help from traditional healers were to get relief for physical health problems affecting the quality of life. Signs and symptoms related to diabetes, expressed as poor glycaemic control, were reported by all participants. Acute or chronic complications may be attributed to few people going to hospital to test regularly for diabetes; most people in Sub-Saharan Africa only do so when the illness has progressed and complications have developed [22]. Symptoms like polydipsia, polyuria and nykturia were mentioned frequently. Micro- and macro vascular complications affecting the eyes and heart were also elicited: impaired vision, high blood pressure and severe headache. In particular, pain in the lower limbs and/or body (head, abdominal) was predominantly reported as an important determinant of traditional medicine use when western medicine seemed not to have helped.

Examining health-care seeking behaviour from a theoretical perspective in this study may further describe the health deviation [15] that is presented as diabetes-related complications. Self-care agency is consequently needed in this case, where self-care and assistance are essential to sustain health. Nurses therefore are greatly needed to offer guidance about therapeutic self-care demands. These demands are tailored to promote supportive education for these clients so that they can adjust to the new lifestyle and master their condition. People with diabetes experience health deviation in self-care and therefore need to seek support and get appropriate medical and nursing assistance. Informative support in managing the disease is particularly important for patients [23]. Nurses are expected to use interpersonal skills in counselling clients with chronic conditions to manage self-care. Untreated pain in these clients demonstrated signs of hopelessness and depression, thus people with poorer health are more likely to be dissatisfied with western treatment [17]. People use traditional medicine mainly because of their own experiences and observations [24] and also reported seeking a cure for diabetes. The findings were in accordance with previous reports on a traditional belief in Uganda that every illness has a cure, unlike scientific medicine with the label of chronic conditions [8, 9].

This study revealed poor individual experience of well-being in relation to complications related to diabetes as the main reason for seeking help from traditional healers. The findings differ from those in a previous study investigating the use of traditional medicine for treating diabetes in Eastern Uganda [9], that traditional medicine is accessible, acceptable, and affordable to meet people's expectations. The latter reported facility-based reasons in health care delivery services, unlike the aforementioned implications of the disease process. Another study in South Africa highlighted a large number of patients with diabetes visiting traditional healers primarily to address social problems, and those from urban areas in particular did this when western medicine did not improve their condition [25]. Inability to adhere to diabetes treatment is a common practice, as it was noted that more than one in every four respondents in Mulago hospital failed to adhere to diabetes treatment in Uganda [5].

Inconsistency in taking prescribed treatment was marked, as some participants stopped taking western medicine and opted for traditional medicine, although most participants mentioned having used western medicine in combination with traditional medicine as complementary medicine. Discontinuity in taking herbal as well as western medicine was noted in some cases when people felt better. This kind of practice was also noted among other patients with other diseases in Congo who stopped taking herbal treatment and it became difficult to monitor their progress [24, 26]. Perceived failure in managing diabetes and effects of western medicine proved to be the determinants for using alternative medicine in the folk sector, consistent with previous studies [6, 25].

The treatment given by the traditional healers was mainly described as unknown extracted medicine in different forms. However, some participants said that it included food, e.g. raw onions, garlic, avocado, guava and mangoes, and medicinal plants or local herbs such as aloe vera and eucalyptus leaves. Some medicinal plants are only for therapeutic use and others are taken as food; for example guava (*Psidium guajava*) has been listed among natural remedies for diabetes [24]. Even though the use of traditional medicine varies widely among different communities, with diabetes commonly practices are the use of nutritional supplements, herbal medicine and nutritional advice [27]. Herbal medicine has been an integral part of traditional Chinese medicine and has been used safely for centuries by trained traditional practitioners [28]. Some studies report that patients who opted to use alternative medicine did so because of perceived reduction of symptoms and lowered costs of side effects associated with western treatment, and patients needed more control of their diabetes [29].

This study highlighted diverse feelings about traditional medicine use to reduce symptoms, and participants needed more control of their condition. However, some patients reported that the treatment had no or unknown effect but hoped it would work and others experienced worsened well-being on complementary medicine. Despite beliefs that herbal medicine entails no risk of harm, practices can be harmful or cause adverse reactions, especially in conjunction with other medicines [7, 30]. An important finding in this study is that many patients did not know what treatment they were given and therefore informing their primary physicians about their CAM usage would be difficult even if they wanted to. This calls for more investigations as well as awareness in staff to ask about this use. Nurses play a key role in assessing data about people's individual beliefs and behaviours [10] including the use of CAM to prevent switching between different caregivers and threatening appropriate treatment [31].

Participants reported many places where they had sought healthcare other than at the facility of traditional healers. Before seeking alternative medicine all participants’ medical diagnoses were made by professionals in government hospitals, health centres and private clinics. This indicates that there might have been a disconnection in the partnership between participants and the former healthcare providers [32]. In Uganda there is no health insurance for patients, and services are underfunded, drugs are frequently unavailable and patients are forced to purchase drugs from other drugstores including CAM practitioners [12]. Further, diabetes clinics have been developed only at some hospitals [10] and this explains the unpreparedness to handle diabetes care within the health care system. Lack of adequate diabetes care could have been one factor influencing people to seek help in different health care facilities including traditional healers. Switching between health care providers may be associated with a high risk of treatment discontinuity that has already been established and consequently affects health [5]. The potential risk of traditional therapies is that they might be used as alternatives to western treatments for serious medical conditions [33]. In this study, those who were on CAM demonstrated having more and severe complications related to diabetes which need to be noted.

The pattern of care seeking behaviour was that most clients simultaneously used the professional health care in government hospitals, healthcare centres and private clinics. Participants who reported having been on complementary treatment mainly went to public hospitals for medical prescriptions and blood glucose tests. Others used all existing health systems: the popular, folk and professional health sectors. However, the findings revealed that most people with diabetes seemed to value western medicine/ conventional treatment but experienced difficulties in engaging continuously in the treatment due to influence from the popular and folk sectors [2], including financial constraints, and individual experience of poor physical well-being.

As regards the influence on decisions to seek health care, this study revealed the popular sector to have played a major role in physical and financial assistance. The family members showed patients where to go, including paying for transport and treatment. Friends and senior patients gave advice from their perceived experience of the treatment. Similarly, recommendations of CAM usage have been shown to be initiated by family members among communities in Australia and Taiwan [27, 29]. Massmedia were another major source of information. Many clients listened to the radio to follow their health providers during outreach days. The lines between the popular, folk and professional sectors function as points of entrance and exist for patients, as described by Kleinman [2]; it has been noted in this study that the popular sector had major part of influence on patients’ decisions to seek health care. Something to note is that the least mentioned source of information was nurses and doctors in the professional health care sector. In summary, the findings of this study support previous findings that subjective ill-health and perceived failure in health care to manage diabetes continues to lead many to seek health care from traditional healers in the folk sector [6].

### Limitations

In demographic characteristics the studied population consisted of persons originating from an area where about three-quarters of the population are living and with a majority not being educated (illiterate) or low educated [20]. Thus, the results might be limited to low educated persons in low socioeconomic position which might influence economy, language use and knowledge, affecting their beliefs and care seeking behaviour. Another limitation might be the inability to generalize the results from a qualitative study. However, the choice of an interactive data collection method like focus-group interviews, carefully analysed, and a thorough description of the studied population is aimed to reach a deeper understanding of the phenomenon under study and gives the reader the possibility to transfer the findings to groups or circumstances with similar characteristics [18, 19]. Thus, the findings help us understand and reflect on different perspectives although they do not explain the reality.

## Conclusion

The pattern of health care seeking described revealed that care was sought from traditional healers in the folk sector as a complement to care from the professional sector in government hospitals, health centres and private clinics, where all had been diagnosed, because of perceived ill-health due to symptoms related to the disease, being signs of poor glycaemic control or complications, wishes for a cure for diabetes, or lack of relief from western medicine. Many had used both western and traditional medicine while some had opted for traditional medicine and used it as an alternative. This mix of different health care sectors and providers entails a risk of inappropriate control of the diabetes. Professionals in the health sector need to be aware of the impact the inconsistent pattern of treatment of diabetes has, and the risk of switches between different health care providers [5], and develop strategies to initiate health promotion interventions to include in the care actors of significance to the patient from the popular, folk and professional sectors, in order to maintain the continuity of effective diabetes care. People take decisions to switch between other treatments without follow-up in the professional health sector. If this inconsistent pattern of treating diabetes persists, the expected outcomes will be too far from those of Hjeim and Atwine^3^ that the onset of diabetes-related complications may prevent or significantly delay their progression with effective treatment.

Individualized professional health care for patients with diabetes has to be initiated. This strategy will create an opportunity for health professionals to get ample time to understand each person's ability to carry out self-management of diabetes after assessment is successfully completed, and to identify whether there are unmet needs and dissatisfaction with delivered care. In the diabetes care team nurses need to play a key role in assessing individual's health beliefs those that affect health-care seeking behaviour and the use of CAM [6] to be discussed with the patient until a desired goal is reached to provide equitable treatment. Health care professionals need to be aware that the popular and folk sectors are good at providing information to patients. Thus, they should intensify health education for patients with diabetes at all levels of the health care system including the lowest level of primary health care.

The complexity of diabetes related to care seeking behaviour calls for intensive health-promotion interventions involving multidisciplinary professionals in the health sector to improve diabetes care in Uganda. The integration of traditional medicine in the formal health system is good because it may promote early referral of patients and might make it easier to follow up patients who opt for alternative medicine. By using both sectors practices that can be harmful or cause adverse reactions, particularly in conjunction with other medications, can be prevented [7] as can switches between different heath care providers [5].
